# In vivo glycerol metabolism in patients with glycerol kinase deficiency

**DOI:** 10.1002/jmd2.12452

**Published:** 2024-09-17

**Authors:** Ankit Shah, Huiting Xu, Hyok Joon Kwon, Fredric E. Wondisford

**Affiliations:** ^1^ Division of Endocrinology, Metabolism and Nutrition, Department of Medicine, Robert Wood Johnson Medical School Rutgers University New Brunswick New Jersey USA; ^2^ University of Arizona College of Medicine Phoenix Arizona USA

**Keywords:** gluconeogenesis, glycerol, glycerol kinase, glycolysis, inborn error of metabolism, mass spectrometry, triglyceride synthesis

## Abstract

Glycerol kinase deficiency (GKD) is an X‐linked recessive disorder due to *glycerol kinase* (*GK*) gene mutations resulting in hyperglycerolermia, hyperglyceroluria, and “pseudohypertriglyceridemia.” In vivo glycerol metabolism has not been assessed in GKD. A 62‐year‐old man with suspected GKD and his extended family underwent whole exome sequencing and fasting blood work with two modes of lipid measurements: (1) standard lipase‐based methodology and (2) nuclear magnetic resonance (NMR). Two overnight fasted men with GKD and a heterozygote female carrier then underwent ^13^C_3_‐glycerol infusion. Affected family members had a novel two‐nucleotide deletion in exon 5 of the *GK* gene (c.373_374del). Compared to their family members (*n* = 14), men with GKD (*n* = 5) had significantly lower total cholesterol levels (3.72 ± 0.70 vs. 4.77 ± 0.85 mmol/L, *p* = 0.024). Compared to NMR, lipase‐based assays overreported triglycerides (5.28 ± 1.38 vs. 0.81 ± 0.32, mmol/L, *p* < 0.001) and underreported low‐density lipoprotein cholesterol values (0.93 ± 0.23 vs. 2.18 ± 0.42 mmol/L, *p* = 0.001) in GKD. Men with GKD could not convert glycerol into glucose or triglycerides, which was preserved in the heterozygote carrier. Glycolytic metabolism of glycerol to lactate persisted in GKD, but it was reduced by a magnitude and, possibly, due to homologous glycerol kinases encoded by other genes. In summary, we report a novel *GK* pathogenic variant; affected men cannot convert circulating glycerol to glucose or triglycerides and have lower cholesterol levels. These results offer a human model for potentially targeting glycerol kinase to treat conditions associated with hyperglycemia and hyperlipidemia and warrant further investigation.

**Synopsis**

In our cohort, men with GKD cannot incorporate glycerol into glucose or triglycerides, have lower circulating cholesterol levels, and have residual glycerol to lactate metabolism; the potential therapeutic benefit of targeting glycerol kinase for hyperglycemia and hyperlipidemia warrants further investigation.

## 1 INTRODUCTION

The enzyme glycerol kinase (GK) phosphorylates glycerol to glycerol‐3‐phosphate, ultimately allowing the molecule to serve as an intermediate for glycolysis and gluconeogenesis.^1^ Additionally, glycerol‐3‐phosphate can enter lipid metabolism by binding to long hydrocarbon molecules to make glycerolipids, including triglycerides, diglycerides, and phospholipids. Given glycerol's importance in both carbohydrate and lipid metabolism, it is no surprise that glycerol kinase is highly conserved across prokaryotes and eukaryotes^2^ and present in multiple tissues, including the liver, kidney, intestine, lymphatics, lung, and heart.^3^

Glycerol kinase deficiency (GKD, OMIM 307030) is a rare X‐linked recessive genetic disorder with an estimated prevalence of one in a million.^4^ The *GK* gene is located on the Xp21.3 locus and has 21 exons. In humans, GKD presents in three distinct forms: (1) the complex infantile form involving multiple contiguous genes on the Xp21 locus and is associated with Duchenne muscular dystrophy and congenital adrenal hypoplasia,^5^ (2) the juvenile form is associated with metabolic acidosis, altered mental status, and hypoglycemia within the first few years of life,^6^ and (3) the adult form is a benign clinical presentation where patients are diagnosed in the setting of “pseudohypertriglyceridemia.”^7^

Most clinical laboratories measure glycerol as an indirect means of reporting serum triglyceride concentrations for routine lipid screening. A lipase enzyme is added to collected serum samples to de‐esterify triglycerides into fatty acids and glycerol molecules; glycerol is then measured as a proxy for triglycerides.^8^ However, patients with GKD have elevated circulating levels of glycerol in their serum (hyperglycerolemia) and are often misdiagnosed with hypertriglyceridemia. To overcome this limitation, laboratory personnel can perform a “blanking” step and subtract the endogenous glycerol before adding the lipase enzyme.^9^ In patients without GKD, this blanking step is unnecessary as the endogenous glycerol concentrations are negligible when measuring triglyceride levels.

Several distinct genetic mutations have been reported to cause GKD, though no genotype–phenotype correlation exists.^4^ While prior studies have assessed glycerol kinase activity in GKD using in vitro methods with fibroblasts and leukocytes collected from patients with GKD,^10^, ^11^, ^12^ there are no in vivo studies. Further, male mice with whole‐body *GK* gene deletion die within the first few days of life, making it difficult to study this disorder.^13^ Affected male neonatal pups show growth delay, hyperglycerolemia, increased serum fatty acid levels, and autonomous corticosterone production, while female heterozygous mice are unaffected.^14^

Here, we report results after isotope tracer infusion of ^13^C_3_‐glycerol to assess whole‐body glycerol metabolism in two men with confirmed GKD and a heterozygote female carrier as well as fasting lipid and carbohydrate biomarkers from them and their family members. We hypothesized that men with GKD cannot metabolize circulating glycerol via gluconeogenic, glycolytic, or triglyceride synthesis pathways and that the heterozygote carrier would have these pathways intact.

## 2 MATERIALS AND METHODS

### 2.1 Genetic testing

Genomic DNA from a buccal swab was enriched for the *GK* gene coding regions and splice junctions using a proprietary targeted capture system developed by GeneDx (Gaithersburg, MD) for next‐generation sequencing with copy number variation calling. The enriched targets were simultaneously sequenced with paired‐end reads on an Illumina platform. Bi‐directional sequence reads were assembled and aligned to reference sequences based on NCBI RefSeq transcripts and human genome build GRCh37/UCSC hg19. After gene‐specific filtering, data were analyzed to identify sequence variants, deletions, and duplications involving the coding exons. ABI sequencing was used to confirm the presence of the variant identified by next‐generation sequencing.

### 2.2 Biomarker testing

Study patients completed overnight fasted blood work at Laboratory Corporation of America (Burlington, NC) for glycemic and lipid biomarkers. Specifically, patients underwent two orthogonal means of serum triglycerides measurement: (1) standard lipase‐based methodology and (2) nuclear magnetic resonance (NMR)‐based.

### 2.3 Tracer infusion

**FIGURE 1:**
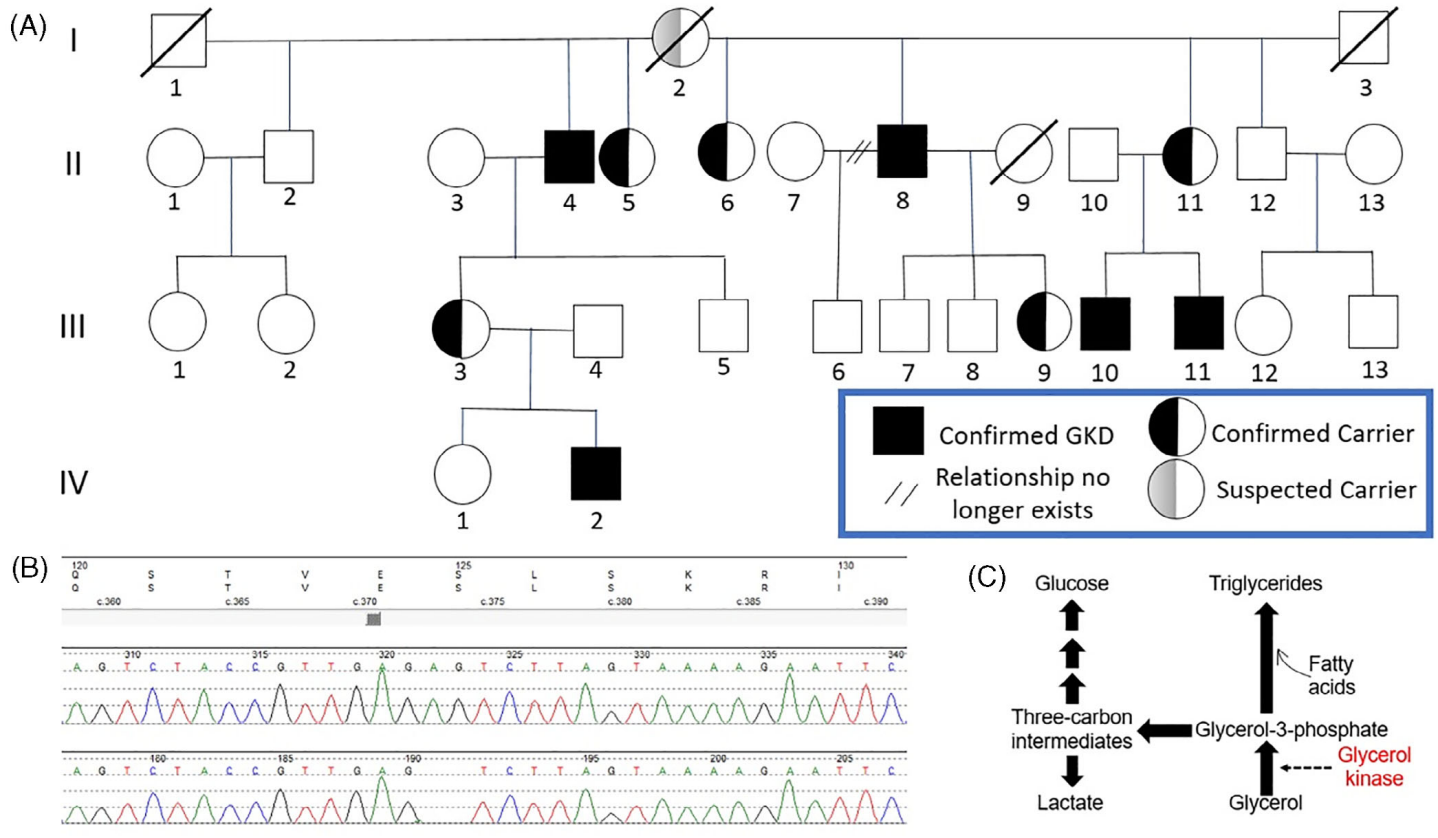
Genetic testing results. (A) Pedigree of an extended family with glycerol kinase deficiency disease. (B) DNA sequencing of the *glycerol kinase* gene from wildtype (top) and affected patients (bottom) showing novel two base pair deletion, c.373_374del, predicted to cause early termination of the protein, p.Leu126*. (C) Schematic of carbon flow from glycerol to glucose, triglycerides, and lactate via glycerol kinase.

On the evening before the study visit, selected patients (II‐8, II‐11, and III‐11, Figure 1A) completed dinner by 8:00 p.m. and began an overnight fast. The patients arrived at the Clinical Research Center (CRC) at Robert Wood Johnson Medical School the following morning at approximately 7:00 a.m. The nursing staff placed an intravenous catheter on each arm; one was for tracer infusion, and the second was for blood collection. At 8:00 a.m. (time = 0 min), the patients underwent an 8‐h infusion of ^13^C_3_‐glycerol (Sigma Aldrich 660702). Given hyperglycerolemia, patients II‐8 and III‐11 received a higher dose of the isotope tracer 2.10 μmol/kg/min, to ensure adequate serum enrichment, while patient II‐11 received 0.75 μmol/kg/min. The latter dose was based on our prior studies in metabolically healthy humans.^15^ No priming bolus was given, and blood and urine were collected before and throughout the tracer infusion.

### 2.4 LC–MS analysis

Serum and urine samples underwent enzymatic derivatization for glucose and glycerol moieties as previously described.^16^ Endogenous serum and urine glycerol concentrations were determined by adding an internal standard of 1 mM glycerol (D_8_‐glycerol, Cambridge Isotope DLM‐558) to the biospecimens. Additionally, serum samples underwent lipid extraction via vortexing with 25:25:50 0.1 M hydrochloric acid:methanol:methyl tert‐butyl ether solution. The top lipid layer was then isolated, dried under air, and resuspended in a 50:50 isopropanol: methanol solution. Prepared samples then went for liquid chromatography‐mass spectrometry (LC–MS) analysis under negative polarity (for glucose, glycerol, and lactate substrates) and positive polarity (for lipidomics) in a stand‐alone orbitrap mass spectrometer.^17^ Analysis was performed on a blank tube to account for background signals. Data were analyzed using El‐MAVEN software suite.^18^ The natural isotope abundances were corrected using AccuCor.^19^

### 2.5 Calculations and statistical analysis

^13^C‐enrichment was calculated on a per‐carbon basis using the formula: [(fraction *M* + 1) + 2 * (fraction *M* + 2) + ⋯ *K* * (fraction *M* + *K*)]/*K*, where *K* is the number of carbon atoms in the metabolite.^20^ Normality was determined using the Shapiro–Wilk test. Paired *t*‐test or Wilcoxon signed‐rank test were used to compare measurements between the two modes of lipid testing. Student's *t*‐test, Mann–Whitney *U* test, or Chi‐squared test were used to compare men with GKD against wildtype and heterozygote family members. All statistical analyses were performed on SPSS (Version 29.0, Armonk, NY) with significance set at *p* < 0.05. Data are reported as mean ± standard deviation, except in figures where they are reported as mean ± standard error.

## 3 RESULTS

### 3.1 Clinical history and family pedigree

The proband, Patient II‐8, is a 62‐year‐old man with no significant medical history who repeatedly had serum triglycerides ranging between 3 and 5 mmol/L (reference <1.70 mmol/L) during routine primary care screening. He carried no risk factors for hypertriglyceridemia, including type 2 diabetes, obesity, alcoholism, or thyroid dysfunction. There was no family history of pancreatitis or premature cardiovascular disease. The physical exam showed a body mass index (BMI) of 19.6 kg/m^2^ and no evidence of cutaneous xanthomas, while a hepatic ultrasound showed no steatosis.

Patient II‐8 saw multiple physicians over several years and was treated with various lipid‐lowering agents, including rosuvastatin, omega‐3 ethyl esters, fenofibrate, gemfibrozil, and niacin. Despite these interventions, the patient's serum triglycerides remained unaltered, raising the treating physicians' concerns about medication noncompliance and undisclosed alcoholism. Ultimately, an academic lipidologist suspected GKD and advised cessation of all lipid‐lowering medications followed by genetic testing. In exon 5 of the *GK* gene, two nucleotides in the 373 and 374 positions were missing, leading to the altered sequence TGAG[delAG]TCTT (Figure 1B).

Patient II‐11 is a 61‐year‐old woman (BMI 19.3 kg/m^2^) with no significant medical history who had normal serum triglyceride levels of 0.80 mmol/L and was found to be a heterozygote carrier. Patient III‐11 is a 28‐year‐old man (BMI 19.9 kg/m^2^) with elevated serum triglycerides at 4.2 mmol/L despite no risk factors and carried the *GK* gene mutation.

### 3.2 Biomarker analysis

**FIGURE 2:**
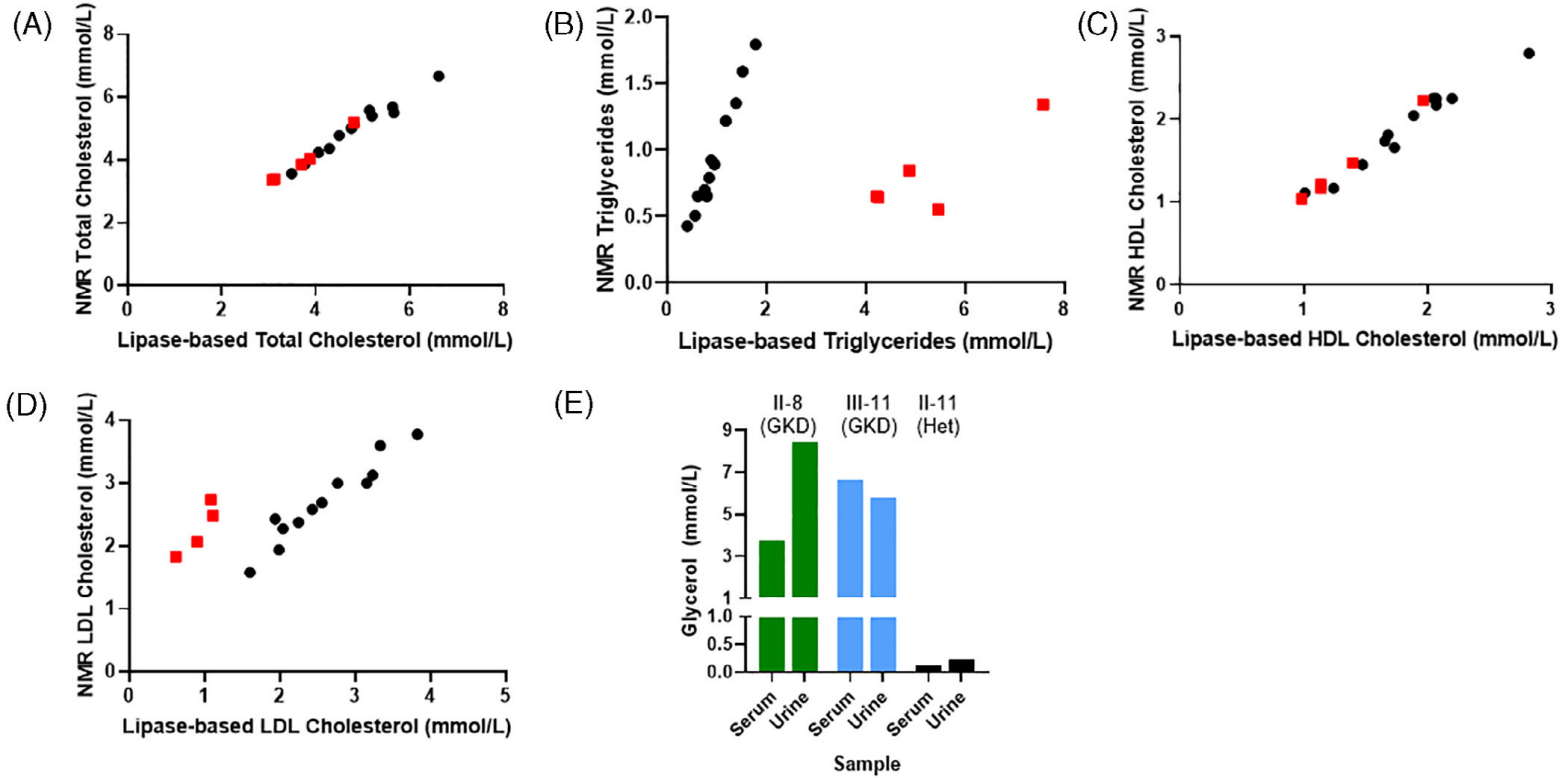
Fasting biomarkers in humans with and without GKD. Serum total cholesterol (A), triglycerides (B), LDL cholesterol (C), and HDL cholesterol (D) between men with GKD (red squares) and related wildtype and heterozygous controls (black circles). (E) Fasting serum and urine glycerol concentrations. Roman and Arabic numbers designate family tree placement in Figure 1A. HDL, high‐density lipoprotein; LDL, low‐density lipoprotein; NMR, nuclear magnetic resonance.

Table 1 shows no differences between patients with GKD and their family members regarding age, body mass index, glycemic control, and circulating free fatty acids. For men with GKD, and compared against NMR, the lipase‐based lipid assay overreported values for serum triglycerides (5.28 ± 1.38 vs. 0.81 ± 0.32, mmol/L, *p* < 0.001) and underreported low‐density lipoprotein (LDL) cholesterol levels (0.93 ± 0.23 vs. 2.18 ± 0.42 mmol/L, *p* = 0.001). Figure 2 shows the correlation between total cholesterol, triglycerides, high‐density lipoprotein (HDL) cholesterol, and LDL cholesterol values by the two modes of lipid testing. Men with GKD were clear outliers from linearity for both serum triglycerides and LDL cholesterol values.

**TABLE 1:** Fasting clinical laboratory data from patients with and without GKD.

	Reference	Hemizygous men with GKD	Wildtype and heterozygous controls	*p* value
*N*		5	14	
Gender (male/female)		5/0	6/9	
Age (years)		42.0 ± 23.5	45.0 ± 18.0	0.770
Body mass index (kg/m^2^)	18.5–24.9	23.1 ± 5.82	25.5 ± 4.28	0.332
Glucose (mmol/L)	3.9–5.5	5.22 ± 0.45	5.08 ± 0.58	0.610
Insulin (pmol/L)	18.1–173	68.1 ± 72.9	65.2 ± 65.3	0.817
HbA1c	<5.7%	5.46 ± 0.36	5.40 ± 0.31	0.935
Free fatty acids (mmol/L)	0.1–0.9	0.40 ± 0.11	0.49 ± 0.16	0.724
Statin use (yes/no)		1/4	1/13	0.421
Total cholesterol (mmol/L)	<5.17	3.72 ± 0.70	4.77 ± 0.85	0.024
Lipase‐based triglycerides (mmol/L)	<1.70	5.28 ± 1.38^a^	0.95 ± 0.39	<0.001
HDL cholesterol (mmol/L)	>1.00	1.32 ± 0.39	1.80 ± 0.48	0.063
LDL cholesterol calculated (mmol/L)	<2.59	0.93 ± 0.23^a^	2.42 ± 0.85	0.004
NMR total cholesterol (mmol/L)	<5.17	3.97 ± 0.75	4.88 ± 0.85	0.049
NMR triglycerides (mmol/L)	<1.70	0.81 ± 0.32	0.96 ± 0.40	0.481
NMR HDL cholesterol (mmol/L)	>1.00	1.42 ± 0.48	1.82 ± 0.49	0.067
NMR LDL cholesterol calculated (mmol/L)	<2.59	2.18 ± 0.42	2.66 ± 0.62	0.059

*Note*: *p* value reflects Student's *t*‐test, Mann–Whitney *U* test, or Chi‐squared comparison between men with GKD and their family members.

Abbreviations: HDL, high‐density lipoprotein; LDL, low‐density lipoprotein; NMR, nuclear magnetic resonance.

^a^ *p* < 0.05 for paired *t*‐test between standard lipid panel measurements versus nuclear magnetic resonance‐based measurements.

Total cholesterol values were significantly lower in men with GKD compared to their family members (Table 1). There were similar trends for reductions in both HDL cholesterol and LDL cholesterol in men with GKD.

### 3.3 Tracer infusion

**FIGURE 3:**
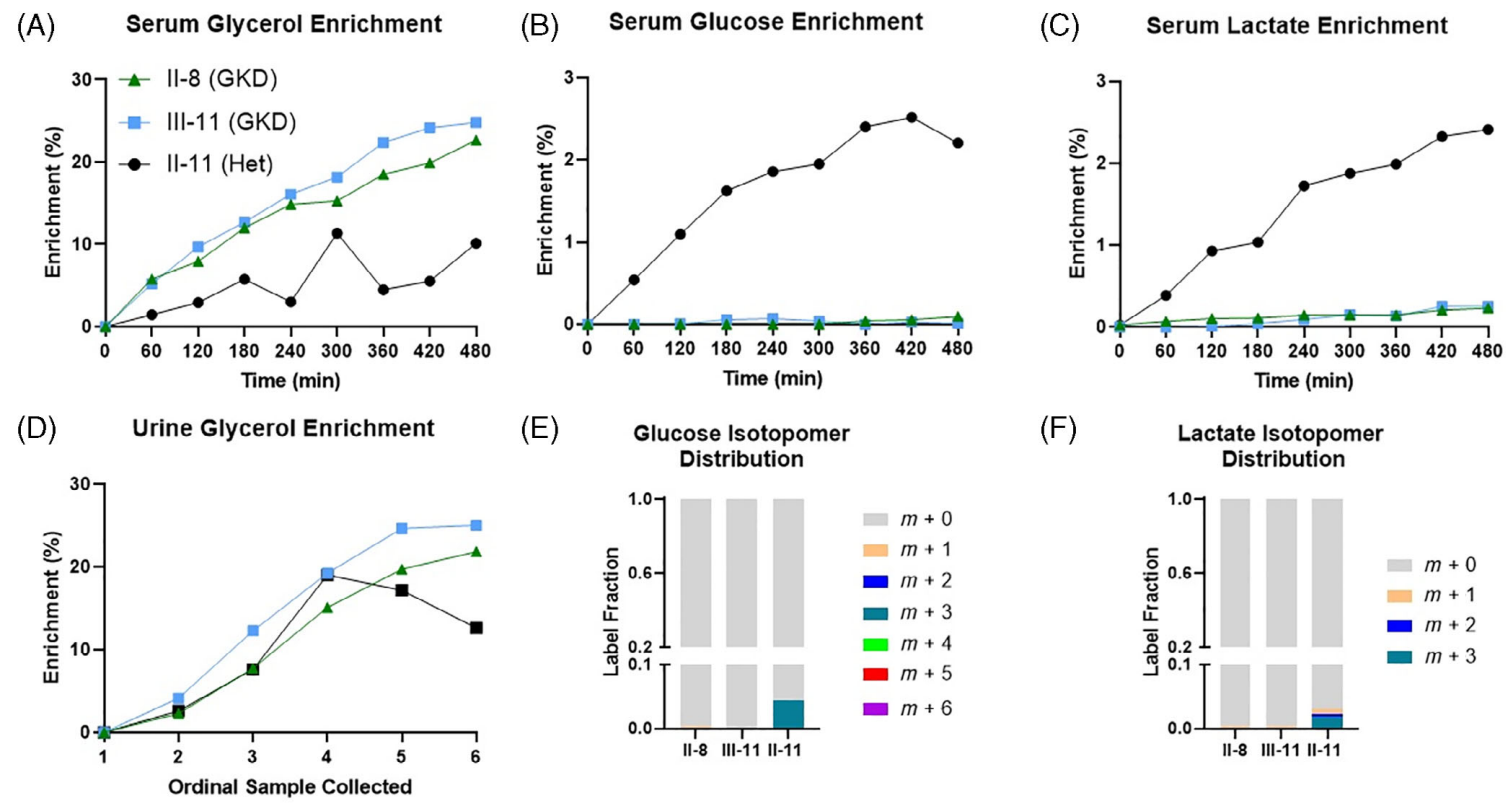
Glycerol‐mediated gluconeogenesis and glycolysis in GKD. ^13^C‐enrichment data of (A) serum glycerol, (B) serum glucose, (C) serum lactate, (D) urine glycerol during ^13^C_3_‐glycerol infusion. Serum glucose (E) and lactate (F) isotopomer distribution from serum collected at final time point. Roman and Arabic numbers designate family tree placement in Figure 1A.

**FIGURE 4:**
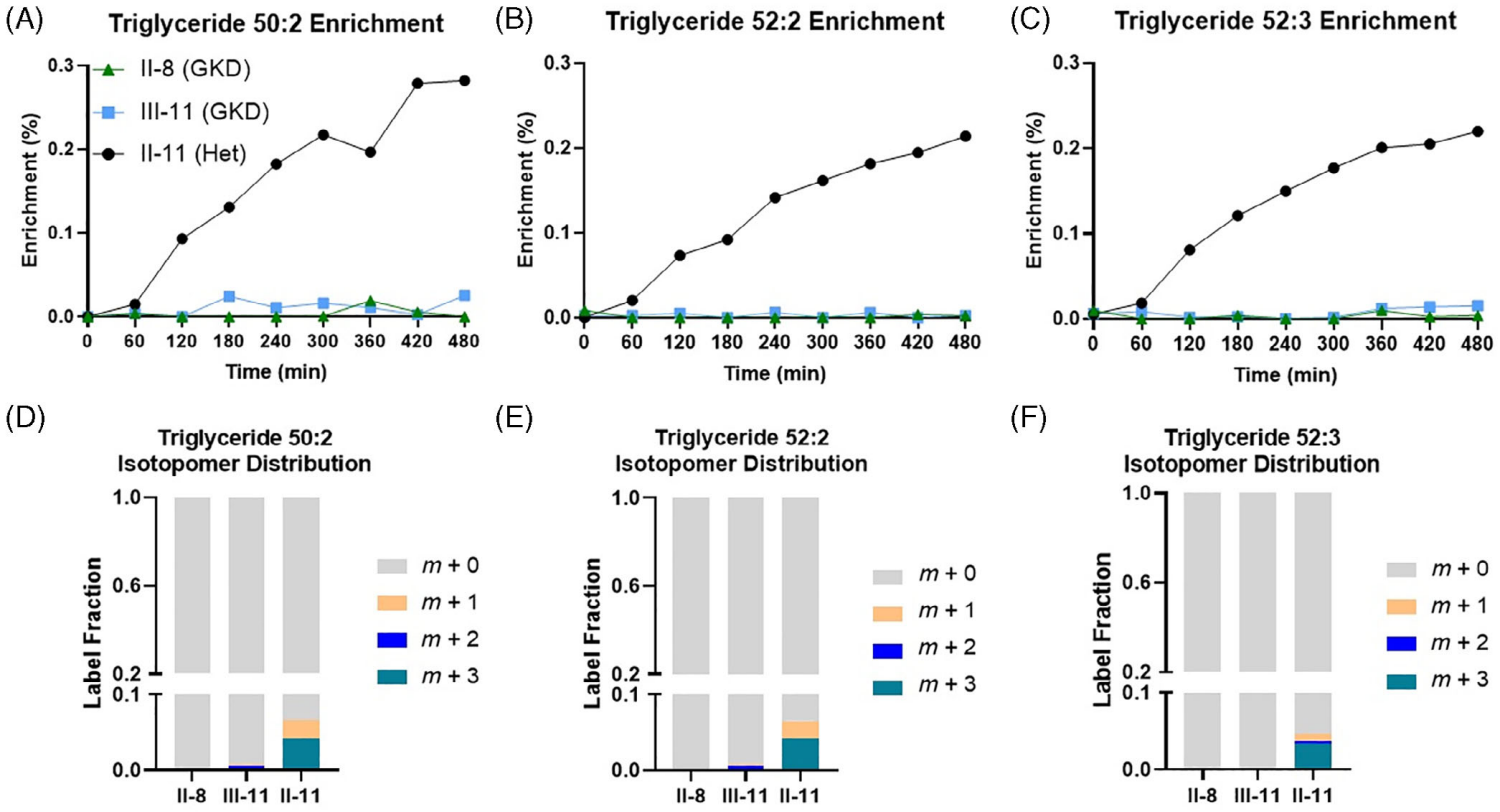
Glycerol incorporation into triglycerides in GKD. Hourly serum ^13^C‐enrichment data of varying triglyceride species during ^13^C_3_‐glycerol infusion (A‐C) and their isotopomer distribution at the final time point (D‐F). Roman and Arabic numbers designate family tree placement in Figure 1A.

The two men with GKD had over a 10‐fold increase in fasting serum and urine glycerol concentrations compared to the heterozygote female carrier (Figure 2E). Figure 3 shows the ^13^C‐enrichment data for serum glycerol (A), serum glucose (B), serum lactate (C), and urine glycerol (D) during the labeled glycerol infusion. The isotopomer distributions of the generated labeled glucose (E) and lactate (F) species are also shown. Figure 4 shows ^13^C‐enrichment data from varying triglyceride species (top) and their respective isotopomer distributions (bottom). Compared to the heterozygous female control, patients with GKD could not convert labeled glycerol to labeled glucose or incorporate the molecule into circulating triglycerides. Patients with GKD can convert glycerol to lactate via glycolysis, though at a much slower rate and lower amount. For the heterozygote carrier, the predominant labeled glucose, lactate, and triglyceride isotopomer species was *m* + 3.

## 4 DISCUSSION

To our knowledge, this is the first report of a deletion of the nucleotides in positions 373 and 374 of the *GK* gene, leading to GKD with predicted nonsense‐mediated mRNA decay and loss of protein function. Patient II‐8's clinical history of being treated with several lipid‐lowering agents with no effective response is a classic example of what can occur in GKD. It often takes an astute clinician to recognize GKD and confirm the diagnosis with a glycerol blank assay or genetic testing. Additionally, one can use a convenient and commercially available NMR measurement of serum lipids to aid in the diagnosis. Detecting GKD can help avoid unnecessary treatment, limit patient anxiety, and screen for other family members who may also be affected.

LDL cholesterol is calculated using the formula total cholesterol minus HDL cholesterol minus triglycerides divided by 5. In patients with GKD, when serum triglycerides are artificially elevated using lipase‐based testing, LDL cholesterol is reflexively underreported, as seen in Table 1 and Figure 2. While pseudohypertriglyceridemia is synonymous with GKD, its impact on the measurement of LDL cholesterol levels has not been appreciated until now. Knowing the accurate values of serum triglycerides and LDL cholesterol, as derived from NMR, can better optimize cardiometabolic care in patients with GKD.

Interestingly, in this family, men with GKD have significantly lower levels of serum total cholesterol driven by comparable reductions in both HDL and LDL cholesterol values. It remains unclear mechanistically why this occurs and whether GKD impacts cholesterol biosynthesis and/or clearance. Using in vitro experiments in rat livers, Migicovsky showed that glycerol prevents the incorporation of the precursor acetate into cholesterol.^21^ He also found that rats given glycerol orally in their drinking water have a reduction of acetate incorporation into liver cholesterol and lower circulating serum cholesterol concentrations. Patients with GKD have high circulating glycerol values, as seen in Figure 2, which can diffuse across hepatic‐specific aquaglyceroporin 9 transporter.^22^ While we only studied serum from our human subjects, others have shown in male mouse pups that *GK* deletion increases cholesterol content in some tissues (brain and muscle) but not all (heart, liver, and kidney) suggesting tissue‐level variability.^23^ Further investigation on the impact of increased circulating glycerol concentrations and altered intra‐tissue glycerol handling on cholesterol metabolism is needed.

Our two patients with GKD cannot convert circulating glycerol to glucose or esterify the molecule to fatty acids to make three triglyceride species that are known to be abundant in the human plasma lipidome.^24^ Gluconeogenesis and triglyceride synthesis occur in the liver, which is a major site for *GK* expression, and so this deficit is not unexpected. Further, our heterozygous female control achieves similar glucose enrichment after ^13^C_3_‐glycerol infusion compared to a cohort of metabolically healthy individuals with normal serum triglycerides, suggesting that one functional *GK* allele is sufficient in heterozygous carriers.^15^ These data parallels findings from female heterozygous mice who are phenotypically normal compared to wildtype counterparts.^14^

Despite their glycerol gluconeogenesis defect, affected members with GKD in this family do not report incidents of hypoglycemia as other substrates, including glycogen, lactate, and amino acids, likely compensate to maintain hepatic glucose production. Similarly, there is no reduction in circulating serum triglycerides as detected by NMR, as other substrates can be used to make the necessary glycerol‐3‐phosphate backbone, including glucose, amino acids, and lactate via glyceroneogenesis. In contrast, *GK* deletion in male mice leads to reduced triglyceride levels in the heart, increased in the brain, and unchanged in the liver, further suggesting tissue‐level specificity related to GK function.^23^ Additionally, these mice have significantly higher levels of circulating free fatty acids, which is not seen in the presented cohort of men with GKD highlighting interspecies variability.^14^

From our heterozygous female carrier, we see the predominant labeled glucose isotopomer species is *m* + 3, suggesting that the fully labeled three‐carbon glycerol molecule wholly converts to glucose. Similarly, the predominant triglyceride species is *m* + 3, suggesting the labeled glycerol provides the backbone for newly formed triglycerides rather than contributing to the carbons on the fatty acid tails. Fatty acid synthesis requires the addition of two‐carbon acetyl‐CoA molecules, meaning the glycerol molecule would need to be broken up, which is not seen.^25^

Contrary to our hypothesis, and to our surprise, our patients with GKD can convert labeled glycerol to labeled lactate via glycolysis. However, it is delayed and is a magnitude smaller than that of the heterozygous carrier. This residual glycerol metabolism may be from homologous proteins. Located on chromosome 4, *GK2* encodes for the protein glycerol kinase 2 and is expressed solely in the testes.^26^ GK2's role in testicular health is an active research topic though prior studies suggest that the enzyme maintains proper mitochondrial health necessary for spermatogenesis.^27^, ^28^ However, prior in vitro assays using murine analogs of glycerol kinase 2 failed to show appreciable enzyme activity.^29^ Additionally, located on chromosome 5, *GK5* encodes for glycerol kinase 5, which is distributed in most, but not all, tissues.^3^ Its physiologic role remains less clear though mouse data exist to support its role in cholesterol biosynthesis in skin tissue.^30^ The exact location(s) of this glycerol to lactate conversion needs further investigation and requires a tissue‐specific approach.

A central pathophysiologic tenet of type 2 diabetes (T2D) is increased gluconeogenesis leading to hyperglycemia.^31^ Gluconeogenesis involves converting noncarbohydrate precursors, including glycerol, into glucose and occurs primarily in the liver (Figure 1C). Glycerol is derived from the lipolysis of triglycerides in adipocytes, and the increased adiposity and insulin resistance associated with T2D enhances lipolysis and glycerol release.^32^ Hepatic *GK* expression also increases in a stepwise fashion in humans with worsening glycemic control from euglycemia to prediabetes to overt T2D.^33^ Thus, a liver in the T2D state has more available glycerol substrate and may be primed to utilize that glycerol toward glucose production. Others have shown glycerol's contribution to gluconeogenesis increases in patients with T2D compared to euglycemic controls.^34^, ^35^

While several distinct medication classes are approved for T2D, none target glycerol metabolism. However, there may be utility in doing so, as glycerol serves as the major carbon source for gluconeogenesis.^36^ Potential therapeutic strategies could include glycerol kinase inhibitors to mitigate hyperglycemia in an insulin‐independent manner. Reassuringly, some forms of GKD can persist in a family over multiple generations, as seen in Figure 1A, with no phenotypic, metabolic, or reproductive consequences. It is hard to gauge whether patients with GKD are protected from T2D when studying isolated families, as other genetic, lifestyle, and dietary factors also have an impact. However, a systematic review of published GKD cases found a lower rate of T2D in affected males with pseudohypertriglyceridemia compared to men with true hypertriglyceridemia.^4^

Typical fasting serum glycerol concentrations in metabolically healthy humans are 0.05–0.1 mM.^37^ In contrast, patients with GKD have supraphysiologic serum glycerol concentrations without apparent clinical impact. In contrast, patients with diabetes mellitus suffer long‐term microvascular and macrovascular complications if circulating glucose levels are two‐ to threefold higher than the physiologic set point. Further, despite the elevated urinary glycerol concentrations in GKD, there is no evidence of nephrotoxicity, in contrast to hyperglycemia‐induced nephropathy. Urinary excretion appears to be a major means of glycerol disposal in GKD, and it is unclear whether these patients have clinically meaningful calorie and weight loss from the hyperglyceroluria.

In summary, we report a novel pathogenic variant within exon 5 of the *GK* gene, leading to the inability of glycerol to contribute to gluconeogenesis or triglyceride synthesis. However, there is minimally preserved glycolytic metabolism of glycerol to lactate, possibly from homologous glycerol kinase proteins. Compared to their family members, affected individuals with GKD have reduced total cholesterol levels with artifactually higher triglycerides and lower LDL cholesterol values from lipase‐based lipid screening. The potential therapeutic benefit of targeting glycerol kinase for hyperglycemia and hyperlipidemia should be further investigated, given the current and increasing prevalence of cardiometabolic diseases globally.

## AUTHOR CONTRIBUTIONS

**Ankit Shah:** Conceptualization, obtaining ethical approval, patient recruitment, study conduct, biospecimen processing, data analysis, and Writing—original draft. **Huiting Xu:** Writing—review and editing. **Hyok Joon Kwon:** Writing—review and editing. **Fredric E. Wondisford:** Conceptualization and writing—review and editing.

## FUNDING INFORMATION

AS was *s*upported by the Patterson Trust Mentored Research Award and Feldstein Medical Foundation. Services, results, and/or products supporting the research project were generated by the Rutgers Cancer Institute of New Jersey Metabolomics Shared Resource, supported, in part, with funding from NCI‐CCSG P30CA072720‐5923. The content is solely the responsibility of the authors and does not necessarily represent the official views of the funding agencies.

## CONFLICT OF INTEREST STATEMENT

The authors declare no conflicts of interest.

## ETHICS STATEMENT

Enrolled subjects underwent informed consent, and the Rutgers University Institutional Review Board approved all study procedures (Pro2022000427). All human subjects research was carried out according to the Declaration of Helsinki.

## Data Availability

The data that support the findings of this study are available from the corresponding author upon reasonable request.
